# Utility of microminipigs for evaluating liver-mediated gene expression in the presence of neutralizing antibody against vector capsid

**DOI:** 10.1038/s41434-020-0125-0

**Published:** 2020-02-17

**Authors:** Ryota Watano, Tsukasa Ohmori, Shuji Hishikawa, Asuka Sakata, Hiroaki Mizukami

**Affiliations:** 1 Division of Genetic Therapeutics, Center for Molecular Medicine, Jichi Medical University, Shimotsuke, Japan; 2 Department of Biochemistry, School of Medicine, Jichi Medical University, Shimotsuke, Japan; 3 Center for Development of Advanced Medical Technology, Jichi Medical University, Shimotsuke, Japan; 4 Division of Cell and Molecular Medicine, Center for Molecular Medicine, Jichi Medical University, Shimotsuke, Japan

**Keywords:** Gene therapy, Imaging

## Abstract

Adeno-associated virus (AAV) vectors can transduce hepatocytes efficiently in vivo in various animal species, including humans. Few reports, however, have examined the utility of pigs in gene therapy. Pigs are potentially useful in preclinical studies because of their anatomical and physiological similarity to humans. Here, we evaluated the utility of microminipigs for liver-targeted gene therapy. These pigs were intravenously inoculated with an AAV8 vector encoding the luciferase gene, and gene expression was assessed by an in vivo imaging system. Robust transgene expression was observed almost exclusively in the liver, even though the pig showed a low-titer of neutralizing antibody (NAb) against the AAV8 capsid. We assessed the action of NAbs against AAV, which interfere with AAV vector-mediated gene transfer by intravascular delivery. When a standard dose of vector was administered intravenously, transgene expression was observed in both NAb-negative and low-titer (14×)-positive subjects, whereas gene expression was not observed in animals with higher titers (56×). These results are compatible with our previous observations using nonhuman primates, indicating that pigs are useful in gene therapy experiments, and that the role of low-titer NAb in intravenous administration of the AAV vector shows similarities across species.

## Introduction

Research on gene therapy using adeno-associated virus (AAV) vectors is currently being conducted worldwide. The AAV vector is derived from a nonpathogenic virus, has a high safety profile, and is capable of transferring genes into a wide range of cells; furthermore, these transgenes can be expressed over long periods of time. Clinical applications for various disease conditions are in progress, and gene therapy successes using AAV vectors have been reported and recently summarized [1].

The liver is a main contributor in several metabolic pathways and a key organ for producing serum proteins. Certain genetic defects in the liver are thought to be the cause of numerous inherited metabolic diseases. Therefore, gene therapy for these defects is expected to provide effective treatments for these diseases. In recent years, liver-directed gene therapy using AAV vectors has achieved clinical improvements in monogenic liver disorders and is likely to be the treatment of choice [2]. Moreover, hemophilia—caused by a single gene mutation—has been one of the best candidate diseases for gene therapy and numerous pioneering studies using AAV have been conducted in relation to this disease [3]. The suitability of this disease for gene therapy lies in the following factors: the therapeutic window of the coagulation factor activity is wide; there is no need to control expression levels; a dramatic therapeutic effect can be obtained by increasing the activity by several percent in severe cases; and a preventive effect can be expected. Effective treatment with persistent transgene expression throughout life using the AAV vector was demonstrated using models of hemophilia B mice and dogs. The success of hemophilia B gene therapy using the AAV8 vector targeting, the liver has been reported [4, 5] and the clinical application of hemophilia B gene therapy has progressed in recent years [6]. Successful cases of hemophilia A gene therapy were also reported in 2017 [7]. These therapeutic strategies targeted the liver, but they can also be applied to other disease conditions; however, the best AAV serotype for human application has yet to be determined.

Although AAV is nonpathogenic, the wild-type virus is widely distributed in nature. Exposure to AAV starts early in life [8–10], with approximately half of the human population testing positive for the neutralizing antibody (NAb) [11–14]. Our interrogation of the Japanese population revealed similar results, showing a positive rate greater than 30% for AAV8 [15]. It is well known that when the AAV vector is injected systemically, the presence of NAb against the AAV capsid inhibits or markedly suppresses the efficiency of gene transfer [16–18]. The negative impact of NAb was observed in a clinical trial wherein two subjects received AAV2 vector (2.0 × 10^12^ vg/kg). One subject had low pretreatment NAb titers to AAV2 (1:2), and expressed peak levels of the coagulation factor IX (FIX) transgene at 12% of the normal level, whereas another subject with a higher pretreatment NAb titer (1:17) did not show any detectable levels of FIX [19], which is similar to the results obtained in subsequent studies in mice and nonhuman primates [20–23]. In the nonhuman primate study, an NAb titer of 1:5 prevented detectable gene transduction of the liver by the AAV8-hFIX vector [20]. Similarly, an NAb titer of 1:4 prevented the equivalent vector dose in severe combined immunodeficiency mice with human intravenous immunoglobulin [21]. We also observed suppression of transgene expression in NAb-positive monkeys [24]. These studies demonstrate the importance of NAb upon systemic AAV vector administration.

Currently, the critical level of NAb titer to affect outcomes is not clearly defined. Therefore, in clinical trials utilizing systemic vector administration, subjects are unanimously excluded from enrollment in cases of pre-existing NAbs [25]. In order to understand the threshold titer of NAb, preclinical experiments using NAb-positive animals are essential. For this purpose, dogs and monkeys have been used as larger animal models alongside rodents. However, due to the rising concern for animal welfare and ethics worldwide, it is increasingly difficult to conduct experiments using these animals. Therefore, the development of nonrodent animal models to replace dogs and monkeys is a socially urgent matter of concern. Pigs, originally limited to use as livestock, have recently been included as experimental models. Moreover, pigs have many similarities with humans in both anatomical and physiological aspects [26]. As the regular-sized pigs are too large to handle (200 kg or larger at maturity), smaller-sized pigs, collectively called as minipigs, have been developed since 1940s, resulting in the establishment of multiple breeds like Clawn, Göttingen, and Yucatan [27]. In recent years, the microminipig was created as the world’s smallest minipig for biomedical research [28]. As their body weight at maturity is ~10 kg, large-scale breeding facilities are unnecessary, smaller amount of reagents are sufficient in experiments, and handling is easy. For these reasons, their usage as well as the number of studies using this animal model is rapidly increasing in various fields including pharmacology [29], gene doping [30], and viral infection [31]. Therefore, evaluating the utility of microminipigs as a new animal model for gene expression is of vital significance. In this study, we tested whether gene expression was observable by intravenous administration of an AAV vector in microminipigs. In addition, we evaluated the threshold titer of NAb related to gene expression.

## Materials and methods

### Cell lines, plasmids, and AAV vector preparation

HEK293 cells purchased from Agilent Technologies (Santa Clara, CA, USA) and 2V6.11 cells obtained from ATCC (Manassas, VA, USA) were maintained as described previously [24].

The AAV8 vector encodes the luciferase gene located downstream of the liver-specific chimeric promoter. It also contains an enhancer element from the hepatic control region (HCR) of the *ApoE/C-I* gene and the 5′ flanking region of the *human α 1-antitrypsin* (HAAT) gene (AAV8-HCRHAAT-luciferase), as described previously [32]. The AAV vector was prepared with the chimeric packaging plasmid (AAV2 rep/AAV8 cap) and the adenovirus helper plasmid, pHelper (Agilent Technologies), as described previously [33]. Titration of the recombinant AAV vectors was performed by quantitative PCR using the real-time PCR system StepOnePlus™ (Applied Biosystems, Tokyo, Japan), as described previously [34].

### Animal experiments

Microminipigs were bred and maintained at the Fuji Micra Co., Ltd. (Shizuoka, Japan). Experiments using the pigs were performed at Jichi Medical University according to the guidelines provided by the Institutional Animal Care and Concern Committees, which also approved all protocols. Invasive procedures were carried out under general anesthesia by inhalation of sevoflurane (Pfizer, Inc., Tokyo, Japan), with vital-signs monitoring conducted in accordance with the stipulated guidelines. Vector solutions were injected into the cervical vein. Microminipigs with low NAb titers (≤56×) were used in this study. To evaluate luciferase expression in pigs, 7 days after vector injection, 15 mg/ml of D-luciferin solution (OZ Biosciences, San Diego, CA, USA) was injected into the unused cervical vein. Animal #1 was then imaged using the IVIS Spectrum CT imaging system (Caliper Life Sciences, Hopkinton, MA, USA).

### NAb assay

The assay for the detection of anti-AAV8 NAbs was performed as previously reported [15]. Briefly, 5 × 10^4^ 2V6.11 cells were seeded into the wells of 96-well culture plates. The serum samples were heat-inactivated at 56 °C for 30 min. On the day of transduction, 10 µl of serum (either undiluted or subjected to twofold serial dilutions with fetal bovine serum) was incubated with the vector (AAV8-CMV-LacZ, 5 × 10^7^ vg/10 µl) at 37 °C for 1 h; this mixture was then added to a culture well. The culture medium was removed after a 48-h incubation, and β-galactosidase activity was quantified with a β-Gal assay kit (Invitrogen, Carlsbad, CA, USA). If β-galactosidase activity of a test sample was inhibited more than 50% of that in control fetal bovine serum, it was considered positive for neutralizing capacity. The inhibitory titer of the serum sample was expressed as the highest final dilution in the culture medium that showed inhibitory activity.

### Quantitation of AAV8 vector DNA in pig tissue

Quantitation of AAV8 vector DNA in pig tissues was performed using quantitative PCR on a StepOnePlus™ instrument (Applied Biosystems). DNA was isolated from pig tissues using a DNeasy Blood & Tissue Kit (Qiagen, Valencia, CA, USA) and subjected to PCR using the following primers:

F: 5′-AGCAATAGCATCACAAATTTCACAA-3′

R: 5′-CCAGACATGATAAGATACATTGATGAGTT-3′

Probe: 5′-AGCATTTTTTTCACTGCATTCTAGTTGTGGTTTGTC-3 (THUNDERBIRD^®^ Probe qPCR Mix; Toyobo, Osaka, Japan).

### Detection of specific anti-AAV8 antibody in serum

Serum anti-AAV8 antibodies were quantified by enzyme-linked immunosorbent assay (ELISA). Briefly, AAV8 capsid was diluted to 1 × 10^8^ vg/µl with 0.01 M phosphate-buffered saline (PBS, pH 7.4). Diluted AAV8 capsid solution (100 µl) was added to each well of a 96-well ELISA plate maintained at 4 °C overnight. On the following day, the plate was washed with PBS and 150 µl of blocking solution (5% BSA) was added to each well and incubated at 37 °C for 2 h. The plate was washed with PBS and 100 µl of serum at a 1:2000 dilution was added and incubated at 37 °C for 90 min. After washing with PBS, 100 µl of rabbit anti-pig IgG antibody tagged with horseradish peroxidase (HRP, 1:5000) (Sigma-Aldrich, St. Louis, MO, USA) was added and incubated at 37 °C for 1 h. After washing, KPL ABTS Peroxidase Substrate System (Seracare Life Sciences, Inc., Milford, MA, USA) was added and incubated for 10 min at 37 °C. The absorbance was quantified with a Benchmark™ Plus microplate reader (Bio-Rad Laboratories, Hercules, CA, USA). Experiments were performed in triplicate.

## Results

**Fig. 1:**
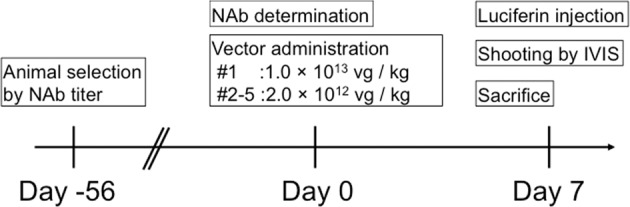
Experimental timeline using microminipigs to observe targeted gene expression in the liver. On day 0, serum was collected from pigs before injection of the vector, the NAb titer against the AAV8 capsid was determined, and the animals used were selected based on their NAb titer. The AAV8-HCRhAAT-luciferase vector was then injected intravenously, and 7 days later, luciferin was injected from the peripheral vein; gene expression was assessed using an IVIS system. Upon animal sacrifice, the number of vector genome copies from each tissue was analyzed using quantitative PCR.

The AAV8 vector carrying a luciferase gene driven by a liver-specific promoter/enhancer (AAV8-HCRHAAT-luciferase) was used to observe transgene expression in microminipigs. The timeline of experiments is described in Fig. 1. Prior to injection of the vector, we selected animals based on their NAb titer. On day 0, serum from each animal was collected just before injection of the vector and the NAb titer against AAV8 capsid was determined. The vector was then intravenously injected. On day 7 post injection, gene expression was assessed using the IVIS system. Upon animal sacrifice, the quantity of vector genomes in each tissue was analyzed using a quantitative PCR assay.

**Fig. 2:**
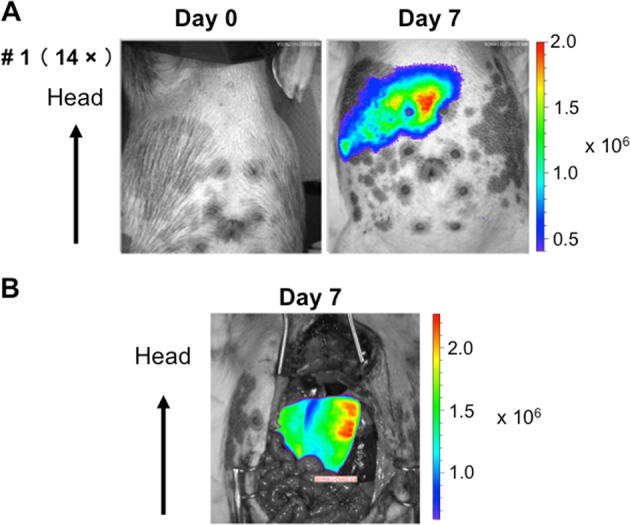
Gene expression in the liver following intravenous administration of the AAV vector. On day 0, a high dose (1.0 × 10^13^ vg/kg) of AAV8-HCRhAAT-luciferase vector was intravenously injected into microminipig #1. On day 7, gene expression was assessed using an IVIS system. **a** Transgene expression was observed in the upper right area of the abdomen. **b** Visceral organs were exposed by an incision in the skin and liver-specific gene expression was observed.

**Fig. 3:**
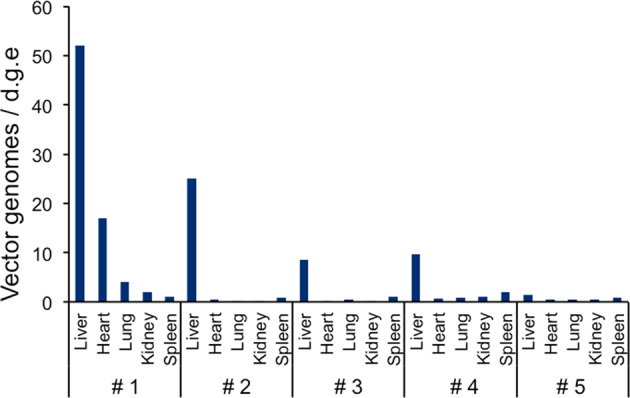
Quantitation and distribution of vector-specific genomes in tissues after intravenous administration of the AAV vector. On day 0, the AAV8-HCRhAAT-luciferase vector was intravenously injected into microminipigs. On day 7, the pigs were sacrificed, and the number of vector genome (vg) copies in representative tissues were determined by quantitative PCR and expressed as copy numbers per cell.

In the first pig, a relatively high dose (1.0 × 10^13^ vg/kg) of the vector was injected. As a result, gene expression was unambiguously observed in the right upper abdominal area (Fig. 2a). Liver-specific gene expression was clearly demonstrated upon skin incision under general anesthesia (Fig. 2b). Following sacrifice, the vector quantity in each tissue was analyzed using quantitative PCR, showing a significant quantity of vector genomes in liver tissue (Fig. 3).

**Fig. 4:**
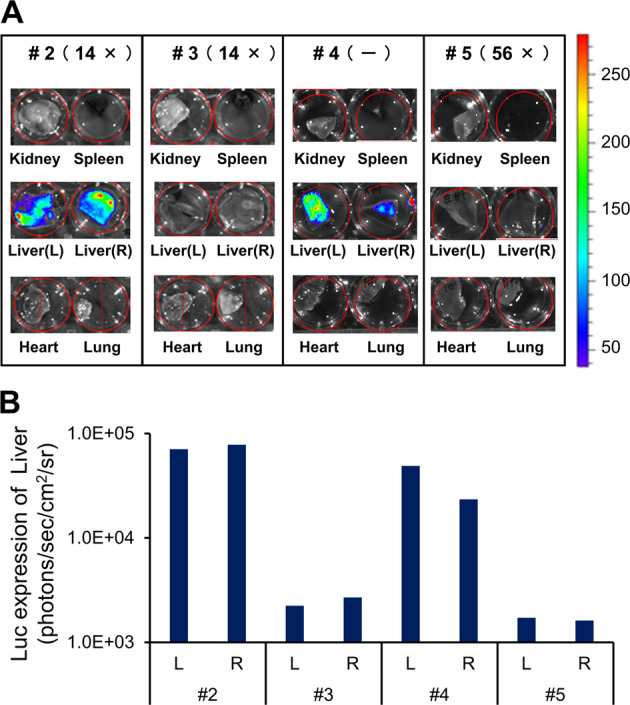
Gene expression in various tissues following intravenous administration of the AAV vector. On day 0, a standard dose (2.0 × 10^12^ vg/kg) of the AAV8-HCRhAAT-luciferase vector was intravenously injected into microminipigs #2–#5. On day 7, gene expression was assessed using an IVIS system and the relationship between the titer of NAb and transgene expression was assessed. **a** Luciferase expression in major organs was assessed by ex vivo experiments. **b** Luciferase expression levels in liver tissues were quantified.

Next, we assessed the action of NAb against AAV. The relationship between the titer of NAb and transgene expression was tested using four pigs. For this purpose, we selected pigs based on NAb titer, from negative titers to 56×. In this series of experiments, a standard dose (2.0 × 10^12^ vg/kg) of the AAV8-HCRHAAT-luciferase vector was injected intravenously and transgene expression was assessed at 1 week. As whole-body IVIS was unavailable at the time this study was conducted, we assessed gene expression mainly via ex vivo experiments. As a result, luciferase gene expression was observed in the livers of both NAb-negative (#4) and minimally positive (14×) pigs (#2), whereas gene expression was not observed in a higher titer (56×) pig (#5) (Fig. 4a**)**. Luciferase expression levels in the livers were quantified and are shown in Fig. 4b.

The copy number of vector genomes in each tissue was analyzed using quantitative PCR. A quantifiable number of vector genomes in the liver was observed in the NAb-negative (#4) and the minimally positive (#2, #3) pigs, whereas the vector sequences were not detectable in the higher titer (56×)-positive (#5) pig (Fig. 3). The expression results for pigs following injection of the AAV8-HCRHAAT-luciferase vector are summarized in Table 1.

**Table 1:** List of pigs used in this study.

Pig number	Weight (kg)	Vector dose (vg/kg)	Anti-AAV8 NAb titer	Luciferase expression^a^	Vector genome copies in liver tissue^b^
#1	2.3	1.0E + 13	14×	(++)	52
#2	5.5	2.0E + 12	14×	(+)	25
#3	5.0	2.0E + 12	14×	(−)	8.5
#4	6.7	2.0E + 12	(−)	(+)	9.6
#5	5.7	2.0E + 12	56×	(−)	1.3

The AAV8-HCRHAAT-luciferase vector was administered intravenously to five microminipigs. All subjects were 3-month-old males at the time of vector administration. One received a higher dose of vector (1.0 × 10^13^ vg/kg) (#1) and the other four pigs received a standard dose (2.0 × 10^12^ vg/kg) (#2–5). The neutralizing antibody (NAb) titer against the AAV8 capsid is expressed as the final dilution of the test serum in the assay. The number of vector genome (vg) copies in liver cells was determined by quantitative PCR and expressed as copy numbers per cell. Luciferase expression was assessed using an IVIS system.

^a^Quantitated results for #2–5 are shown in Fig. 4b.

^b^Copy numbers are shown as vg/diploid genome.

**Fig. 5:**
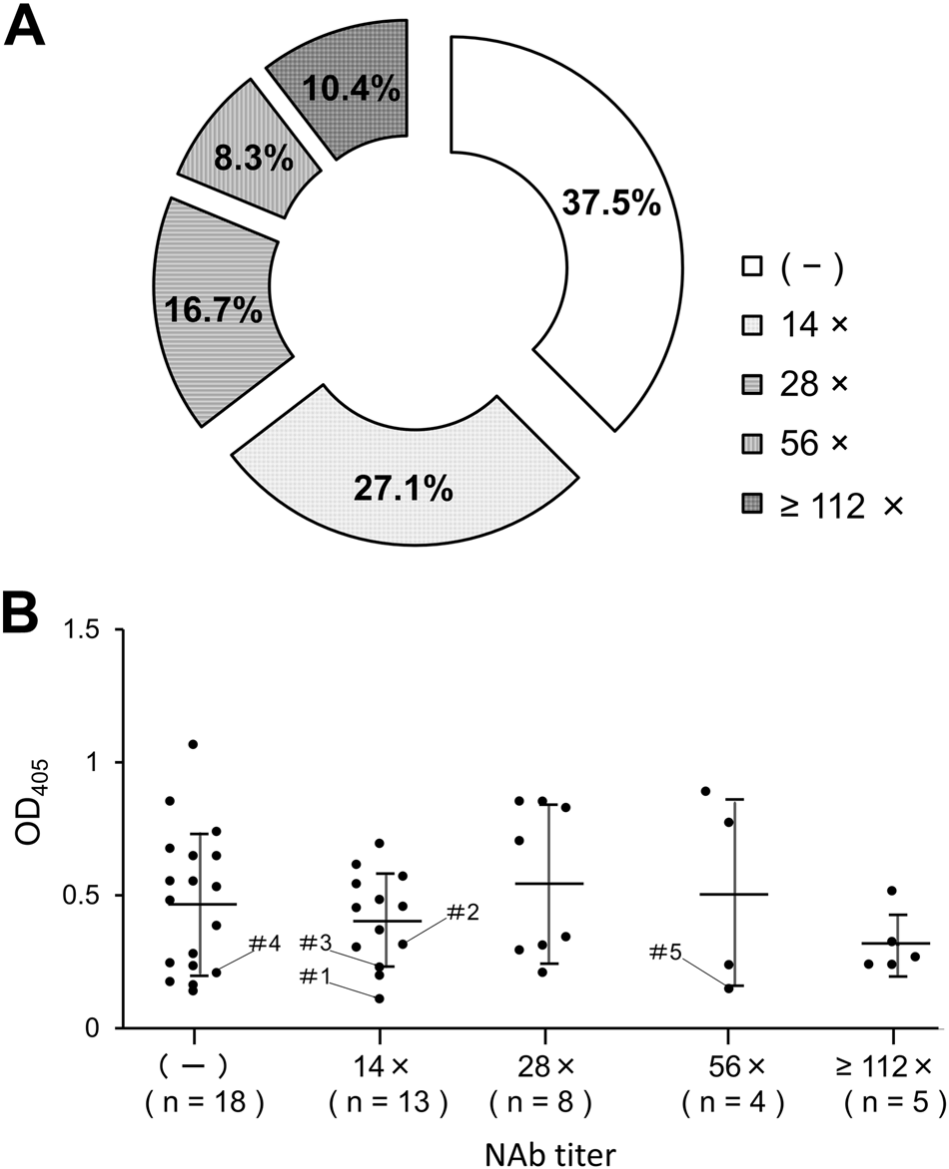
Titers of NAb against AAV8 and the amount of anti-AAV8 IgGs in microminipigs. Blood samples were collected from microminipigs (*n* = 48) from a single breeding farm. Serum was prepared from blood samples and stored at −20 °C until measurement. **a** The anti-AAV8–neutralizing antibody (NAb) titer is expressed as the final dilution of the test serum in the assay. **b** The level of specific anti-AAV8 immunoglobulins present in the serum was evaluated by ELISA. The relationship between the amount of anti-AAV8 antibody and NAb titer was analyzed. Dilution titer of the serum was 1:2000. Data are shown as mean ± SD. One-way ANOVA test was used to determine statistical significance.

In order to evaluate the overall utility of the microminipig, we examined the prevalence of NAb against AAV8 capsid. Blood from 48 pigs in the same facility were collected and their NAb titers were analyzed. As a result, the overall NAb positivity was 62.5%. The proportion of animals with NAb titers of 14×, 28×, 56×, and 112× (or more) was 27.1%, 16.7%, 8.3%, and 10.4%, respectively (Fig. 5a). Moreover, the quantity of anti-AAV8 antibodies (IgG) in pig sera was tested by ELISA and its relationship with NAb titer was analyzed, showing no statistical significance between these two parameters (Fig. 5b).

## Discussion

Recombinant AAV vectors are used to transfer therapeutic genes to target organs, including the liver. To date, liver-directed gene expression has been extensively studied in mice, dogs, and primates—including humans—but not in pigs. In this study, we first confirmed transgene expression in pigs using an IVIS system. To the best of our knowledge, this is the first study to demonstrate liver-mediated transgene expression using an in vivo imaging system in a pig. We then tested expression profiles of animals with various NAb titers. These results indicated that microminipigs may be a good model candidate for gene therapy experiments. The microminipigs can be exported to international locations (Fuji Micra Inc. Homepage), as many other commercially available minipigs for biomedical research.

The presence of NAbs is a recognized problem when administering AAV vectors from peripheral veins. The negative impact of NAb on gene expression has been reported in humans, monkeys, and mice [19–21, 24]. In this study, we demonstrated that the presence of low-titer NAb affects transgene expression in vivo, and that the action of low-titer NAb on gene expression may be similar across species.

We clearly demonstrated that transgene expression in the liver was observed in both NAb-negative and minimally positive (14×) subjects, but not in a subject with a higher titer (56×). These results indicate that the threshold NAb value for transgene expression in our experiment lies near the minimally positive animal, at a standard vector dose (2.0 × 10^12^ vg/kg). In our previous study using nonhuman primates, no transgene expression was observed in any of the three animals with minimally positive (14×) NAb titers [24]. Therefore, there is a possibility that in microminipigs, the threshold level of NAb may be slightly higher than that in primates. Clearly, increasing the vector dose may overcome the inhibitory action of NAb, as observed in our experiment (subject #1). Nonetheless, the relationship between vector dose and NAb level is still not well understood and must be investigated further. The presence of a minimal level of NAb may affect gene expression; therefore, the majority of patients with positive NAb titers cannot be candidates for intravenous vector administration. Therefore, it is important to develop methods to bypass the suppressive action of NAb [35]. Various strategies have been proposed to overcome NAb, such as the use of balloon catheters followed by saline flushing [24], administering empty capsids to adsorb NAb as a decoy [36], and using exosome-enveloped AAV vectors [37]. However, clinically relevant methods have not been established and further study is needed.

In this study, variability in gene expression was observed between subjects with NAb titers of 14× (#2, #3) and in the NAb-negative subject (#4), suggesting differences in gene expression. There was a difference in expression between #2 and #3, despite the fact that their NAb titers were the same. There may be a number of factors contributing to this difference. Primarily, it can be explained by the current system of antibody titration. The NAb titer is usually described by the dilution factor; a subject labeled with a factor of 14× may actually have a titer between exactly 14× and just below 28×, spanning nearly twice the range within the same titer group. To evaluate the effect of NAb in greater detail, a more precise method for describing NAb may be necessary. Other factors may also contribute to the aforementioned differences. For example, the hemodynamic status of individual animals under general anesthesia at the time of vector and luciferin administration (#4 experienced transient cardiovascular weakness at the time of expression analysis) may affect the degree of gene expression. In previous reports, variations in gene expression were observed even when the same amount of vector was injected [4, 5, 20, 21, 24], which was also observed in monkeys and mice [20, 21].

Variability was also observed in the number of vector-derived genome copies in the livers of both NAb-negative (#4) and minimally positive subjects (#1, #2, #3). Gene expression and a measurable number of vector genomes in the liver were observed in the NAb-negative subject (#4). On the other hand, gene expression was not observed in the minimally (14×) positive subject (#3), although a measurable number of vector genomes in the liver was confirmed. Overall, there appears to be a specific relationship between expression of the luciferase gene and the number of vector genomes in the liver (Table 1). In this experiment, the lower limit of luciferase gene expression correlated with ~10 vector genomes/cell. The low levels of transgene expression in this series of experiments may be due to the relatively short observation period of 1 week—a definite constraint in our facility at the time of the experiment. At 1 week, although luciferase expression was observable, the expression level had not yet reached a plateau, resulting in an expanded variance of transgene expression. One may consider the possibility that the results are influenced by the antibodies against the current transgene. However, this is unlikely because 1 week constitutes insufficient time in which to develop an antibody in vivo; furthermore, as luciferase is expressed within the cellular membrane, the antibody cannot affect the expression status developed within that timeframe.

Along with the advantages described above, the pig shows another merit in gene therapy research in that the positivity of NAb against AAV8 was 62.5% and the proportion of low-titer-positive subjects was high. The proportion of NAb-negative and low-titer (14×)-positive subjects was 37.5% and 27.1%, respectively (Fig. 5a). Therefore, microminipigs may be suitable to test the role of low-titer NAb in liver-targeted gene therapy and ultimately useful for evaluating the role of NAb in preclinical studies. Finally, our results show that quantitating anti-capsid antibodies by ELISA did not predict the titer of NAb (Fig. 5b). ELISA reflects the amount of overall IgG bound by the AAV8 capsid, whereas the NAb indicates the inhibitory action. It is not clear to what extent ELISA and the NAb titer correlate. One study reported little correlation in AAV2 and AAV8 [38], while in another paper, a correlation was suggested in AAV5 with a broad range of NAb titers [39]. As only low NAb titer animals were involved in our study, it may have been difficult to observe the correlation between these parameters.

In conclusion, we demonstrated targeted gene expression in pig liver and determined the threshold level of NAb titer by intravenous AAV8 vector administration. Our results provide valuable insight for using microminipigs in preclinical trials for liver-targeted AAV gene therapy in the future.
